# Mesenchymal Stem Cells Loaded in Injectable Alginate Hydrogels Promote Liver Growth and Attenuate Liver Fibrosis in Cirrhotic Rats

**DOI:** 10.3390/gels11040250

**Published:** 2025-03-27

**Authors:** Dominic Karl M. Bolinas, Allan John R. Barcena, Archana Mishra, Marvin R. Bernardino, Vincent Lin, Francisco M. Heralde, Gouthami Chintalapani, Natalie W. Fowlkes, Steven Y. Huang, Marites P. Melancon

**Affiliations:** 1 Department of Interventional Radiology, The University of Texas MD Anderson Cancer Center, Houston, TX 77030, USA; dkbolinas@mdanderson.org (D.K.M.B.); ajbarcena@mdanderson.org (A.J.R.B.); archanamishra56@gmail.com (A.M.); mrbernardino@mdanderson.org (M.R.B.); vincentlin2006@outlook.com (V.L.); gouthami.chintalapani@siemens-healthineers.com (G.C.); syhuang@mdanderson.org (S.Y.H.); 2 College of Medicine, University of the Philippines Manila, Manila 1000, Philippines; fmheralde1@up.edu.ph; 3 Department of Veterinary Medicine and Surgery, The University of Texas MD Anderson Cancer Center, Houston, TX 77030, USA; nwfowlkes@mdanderson.org; 4 The University of Texas MD Anderson Cancer Center UTHealth Houston Graduate School of Biomedical Sciences, Houston, TX 77030, USA

**Keywords:** alginate, hydrogel, mesenchymal stem cells, liver cirrhosis

## Abstract

Cirrhosis, a marker of severe liver diseases, limits future liver remnant (FLR) growth, preventing many cancer patients from undergoing surgery. While portal vein blockade (PVB) techniques are used to stimulate liver regeneration, 20–30% of patients still fail to achieve the required growth. Although mesenchymal stem cell (MSC) therapy improves PVB, its efficacy is limited by poor cell retention. To address this, we utilized alginate hydrogels to deliver MSCs and improve their retention. MSCs were loaded in the hydrogel and injected intraportally in cirrhotic rats. Liver volume, weights, enzyme levels, and histology were monitored. Results showed that the hydrogel maintained 89.0 ± 3.0% cell viability and gradually released MSCs for over two weeks. Furthermore, the rats injected with the MSC-loaded hydrogel demonstrated higher liver volumes (FLR ratio of 0.57 ± 0.32) and weights (FLR ratio of 0.84 ± 0.05). The treated rats exhibited more improved liver enzymes (AST: 72.75 ± 14.17 U/L, ALP: 135.67 ± 41.20 U/L, ALT: 46.00 ± 2.94 U/L) and decreased fibrotic areas in the liver (4.52 ± 0.22%) compared to the control group. Histology revealed increased retention when MSCs were delivered with the hydrogel (37.30 ± 16.10 MSCs/mm^2^) compared to cells alone (21.70 ± 22.10 MSCs/mm^2^). Overall, the MSC-loaded hydrogels enhanced the growth and reduced the fibrosis of the liver by promoting cell retention and efficacy in cirrhotic rats. This approach holds significant potential for improving outcomes among cancer patients, offering a promising therapeutic strategy for liver regeneration and treatment of liver diseases.

## 1. Introduction

Cirrhosis is a hallmark of all chronic liver diseases, affecting 122 million people and causing about two million deaths globally [1,2]. It is the most potent risk factor for liver malignancies (i.e., 80% to 90% of patients with hepatocellular carcinoma have cirrhosis) [3]. The high degree of underlying cirrhosis prevents patients from undergoing liver resection because it limits the regenerative capacity of the future liver remnant (FLR) [4]. In cirrhotic livers, the minimum FLR volume for adequate recovery is at least 40% of the total liver, which is higher than the cutoff of 25% in normal livers. A considerable number of patients do not meet these thresholds and become unsuitable for curative resection because of higher risk for post-hepatectomy liver failure [5,6]. In an attempt to induce FLR growth, preoperative portal vein blockade (PVB) (e.g., portal vein ligation (PVL) or portal vein embolization (PVE)) is performed before major liver resection [7]. Despite this, 30% of patients still fail to undergo curative resection. The cirrhotic changes in the liver hinder the effectiveness of PVB, causing slow and insufficient FLR growth [8,9].

Current research explores the application of mesenchymal stem cells (MSCs) for ameliorating liver cirrhosis [10]. More than 80 clinical trials using MSCs for liver diseases are showing promising results in improving liver function and histological grade [11]. The therapeutic potential of MSCs is largely attributed to their ability to differentiate into hepatocyte-like cells [12] and their capability to produce regenerative growth factors and immunomodulatory cytokines [13,14]. Because of this, MSC therapy is explored as an augmentative strategy for PVB to achieve maximal volumetric gain in a short amount of time [15]. However, direct injection of MSCs results in problems such as low survival and low retention of the cells within the liver. MSCs lose their efficacy due to prolonged exposure to a suboptimal microenvironment and the loss of supporting extracellular matrix (ECM) [16,17]. The absence of established techniques and technologies to support MSC delivery limits their therapeutic use in liver diseases.

Alginate biomaterials, with their superior biocompatibility, biodegradability, and ability to closely mimic the natural ECM, are increasingly being recognized for their potential as cell delivery systems [18]. Alginate is a natural polymer that does not degrade to toxic byproducts [19,20], as compared with some synthetic polymers [21,22]. They can form injectable hydrogels, comprising a three-dimensional network of polymers that contain a large amount of water [23]. Recent studies have shown that hydrogel biomaterials possess bioactive properties beneficial for MSC growth and function [24,25,26]. Alginate hydrogels are explored as MSC delivery systems to treat many diseases, including liver cirrhosis [27,28,29]. They protect MSCs from the hostile environment of the diseased liver, reducing immune-mediated clearance and prolonging cell survival [30]. Furthermore, alginate hydrogels sustain the release of paracrine factors that promote tissue repair and reduce fibrosis in the liver [31]. The hydrogel shields the cells from harmful mechanical forces and acts as a scaffold for local cell adhesion. This allows for controlled intraportal delivery and enables MSCs to directly enter the liver [32].

Although previous studies have explored the potential of MSCs for liver cirrhosis, little emphasis has been placed on improving techniques for efficiently delivering and retaining these cells in the liver following PVB. Considering the outstanding properties of hydrogels in the existing literature, we hypothesized that alginate hydrogels could enhance the retention and therapeutic effects of MSCs, promoting liver regeneration and attenuating fibrosis. In this study, we explored the use of alginate as an injectable hydrogel delivery system to improve MSC retention and function in a fibrotic rat model following portal vein blockade. To the knowledge of the researchers, this is a pioneering study in exploring a biopolymeric scaffold designed to enhance MSC survival and efficacy in this context.

## 2. Results and Discussion

### 2.1. MSC Loading in Alginate Hydrogels

**Figure 1:**
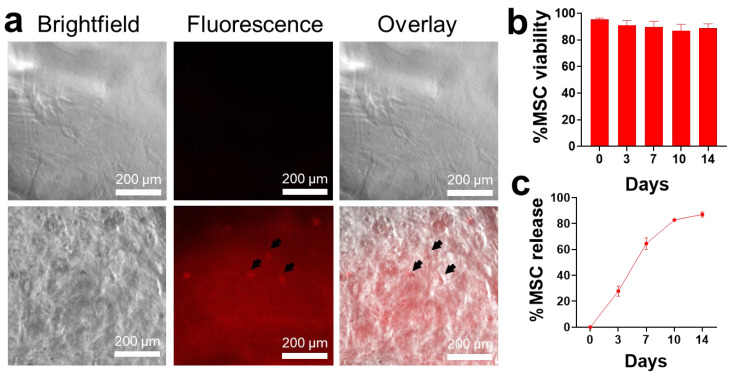
Characterization of MSC-loaded hydrogels. (**a**) Brightfield (left), fluorescence (middle), and overlay (right) images from microscopy show wide distribution of RFP-MSCs (black arrows) within the hydrogel scaffold. (**b**) Viability of the MSCs was maintained after loading in hydrogels (quantified through trypan blue staining). (**c**) MSCs were gradually released over 14 days. Abbreviations: RFP-MSCs, red fluorescent protein–mesenchymal stem cells.

MSCs were loaded in alginate prior to the addition of calcium chloride (CaCl_2_) to form hydrogels. The resulting mixture produced an amorphous, semi-solid hydrogel containing the cells. The swelling study showed 6.08 ± 1.62% and 7.25 ± 2.48% for alginate hydrogels and MSC-loaded hydrogels, respectively (Supplementary Figure S1). Fluorescence microscopy revealed a wide distribution of fluorescent-labeled cells within the matrix system, as seen in Figure 1a. To verify the viability of MSCs after loading, the hydrogel samples were dissolved every 3 days and tested using the trypan blue assay. Over 14 days, the MSCs grown in the hydrogel scaffold remained viable (89.0% ± 3.04) (Figure 1b). Alginate hydrogels may have acted as a supportive matrix for cells due to their inherent biocompatibility and nontoxicity [18]. Pangjantuk et al. demonstrated a high survival rate of human MSCs (77.36%) when loaded in alginate-based hydrogels for a two-week culture period, similar to the findings of this study. The researchers attributed the MSC survival to the increased expression of genes related to stemness (*OCT-4*, *NANOG*, *SOX2*, and *SIRT1*), tissue growth and development (*YAP* and *TAZ*), cell proliferation (Ki67), and telomere activity (*hTERT*) [33]. Other studies also reported similar findings of high cell viability for two weeks using a composite of gelatin–alginate hydrogel scaffold [34] as well as alginate microcapsules [35]. For future studies, understanding cellular behavior and molecular interactions with the hydrogel is a key consideration to further evaluate the role of alginate as a supportive matrix.

The release mechanism of the hydrogels within two weeks was also evaluated. The results showed a gradual release of MSCs over the period. About 20–30% of cells were released every three days, suggesting the gradual degradation of the alginate hydrogels. By the end of the experiment, the majority (86.0 ± 1.61%) of the cells were released (Figure 1c). Our study highlighted the ability of the hydrogel to hold cells in vitro, potentially preventing rapid washout in vivo. Moreover, alginate hydrogels may have allowed the sustained release of cells and their associated therapeutic factors at the desired target tissue, as demonstrated in other studies [36,37]. Overall, the findings showed that MSCs can be loaded in an injectable alginate hydrogel format, which is important for future applications in the minimally invasive delivery of MSCs into the liver [38].

### 2.2. Establishing a Rat Model for Cirrhosis and Portal Vein Ligation

**Figure 2:**
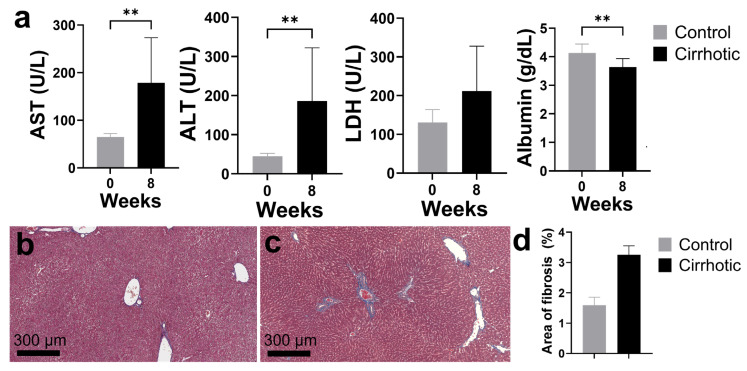
Functional and histologic changes in cirrhotic rat models. (**a**) Serum chemistry levels of liver damage markers (AST, ALT, and LDH) and synthetic function (albumin) showed a significant increase after induction of cirrhosis. (**b**–**d**) Trichrome staining of liver samples from (**b**) control vs. (**c**) cirrhotic rats showing increase in the collagen staining and fibrosis (**d**) Abbreviations: AST, aspartate aminotransferase; ALT, alanine aminotransferase; LDH, lactate dehydrogenase; ** *p* < 0.01.

To properly evaluate MSC therapy, animal models that adequately simulate fibrotic liver disease should be utilized. Different induction techniques (e.g., hepatotoxic chemicals, surgical bile duct ligation, gene knockouts, etc.) have been explored to replicate the major features of liver cirrhosis in murine models [39,40,41,42]. In the current study, intraperitoneal injection of carbon tetrachloride (CCl_4_), a hepatotoxic substance, was used to induce cirrhosis. For a period of 8 weeks, the rats were intraperitoneally injected with CCl_4_. Alterations in the levels of liver markers were observed in the rats after induction (Figure 2a). There were significantly elevated levels of hepatocyte injury markers such as alanine aminotransferase (ALT; from 44.43 ± 7.87 U/L to 185.80 ± 136.40 U/L), aspartate aminotransferase (AST; from 64.86 ± 7.13 U/L to 178.60 ± 94.72 U/L), and lactate dehydrogenase (LDH; from 130.40 ± 33.57 U/L to 211.50 ± 116.50 U/L). Meanwhile, albumin levels, a marker of liver synthetic function, decreased (from 4.11 ± 0.31 g/dL to 3.64 ± 0.30 g/dL). Similarly, another study [43] demonstrated elevated levels of serum ALT and AST and decreased albumin in CCl_4_-treated rats with associated fibrosis deposition. Boyd [44] reported that LDH levels had the highest increase in rats where there was extensive hepatic necrosis. LDH is a cytoplasmic cellular enzyme that is used to detect cell damage or death [45]. This demonstrates that the CCl_4_-treated rats are suitable models for evaluating experimental treatment.

Liver tissue samples were stained with trichrome dye to visualize collagen deposition, an indicator of fibrosis (Figure 2b,c). Figure 2d illustrated the increase in the fibrotic areas (blue staining) in CCl_4_-treated rats (3.25 ± 0.28%) compared to the control (1.59 ± 0.24%). Qualitatively, the staining was more extensive in the periportal areas with septal formation. These results show that the CCl_4_ treatment leads to chronic liver injury and deposition of fibrotic markers. Likewise, Fortea and colleagues [46] reported marked increase in areas of fibrosis (2.83%) in CCl_4_-treated rats. CCl_4_ is metabolized by the cytochrome P450 enzymes and converted to highly reactive trichloromethyl-free and peroxyl radicals that induce lipid peroxidation and cellular damage [47]. Repeated administration of CCl_4_ leads to centrilobular necrosis, portal hypertension, and activation of hepatic stellate cells, causing ECM deposition and elevation of liver enzymes [43]. These lead to pathological effects consistent with the increased collagen and liver function changes observed in the study. These closely resemble the biochemical, histological, and hemodynamic alterations seen in human patients [48].

**Figure 3:**
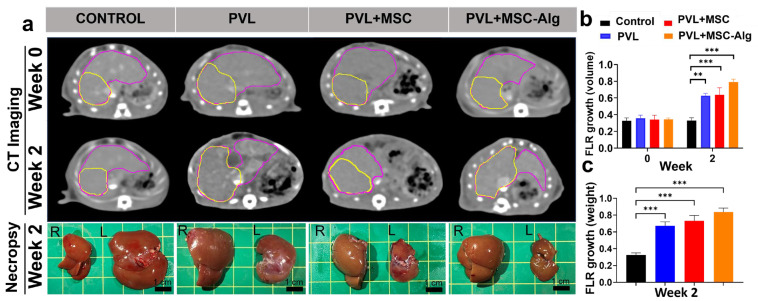
Future liver remnant (FLR) growth by CT volume and weight in CCl_4_-treated rats. (**a**) CT imaging showed an increase in FLR volume (yellow) relative to total liver volume (violet) in the treatment groups, with greatest growth observed in the MSC-loaded hydrogel group. Visual inspection of the gross livers also showed notable increase in FLR in the MSC-loaded hydrogel group compared to the control. To assess the degree of growth, the FLR-to-total liver ratio was quantified by both volume (**b**) and by weight (**c**) across the different treatment groups. ** *p* < 0.01; *** *p* < 0.001. Abbreviations: Alg, alginate; CT, computed tomography; FLR, future liver remnant; MSC, mesenchymal stem cell; PVL, portal vein ligation.

Two weeks after treatment, the FLR growth (non-ligated lobe) was measured using volumetry (in computed tomography) and gross necropsy of the rat livers (Figure 3a). Our study utilized imaging and volumetry studies to monitor liver growth changes in vivo. Clinically, liver volumes are used as a prognostic factor of treatment success. Volumetry is the measurement of the liver volumes performed by manual tracing of the liver boundary and summation of liver area on each section of a CT scan [49]. In this study, volumetry (Figure 3b) revealed an increase in liver volume after two weeks in all treatment groups relative to the control group (control: 0.33 ± 0.00, PVL: 0.49 ± 0.19, PVL+MSC: 0.49 ± 0.21, PVL+MSC-Alg: 0.57 ± 0.32). Indeed, the volumes correlated well with the liver weights (Figure 3c; control: 0.32 ± 0.03, PVL: 0.67 ± 0.05, PVL+MSC: 0.73 ± 0.06, PVL+MSC-Alg: 0.84 ± 0.05). This illustrates the potential of non-invasive in vivo imaging techniques in monitoring the liver and their role in developing predictive models for liver growth and for monitoring treatment response. Importantly, the PVL with MSC-loaded alginate hydrogels group demonstrated the highest increase in both CT and necropsy.

**Figure 4:**
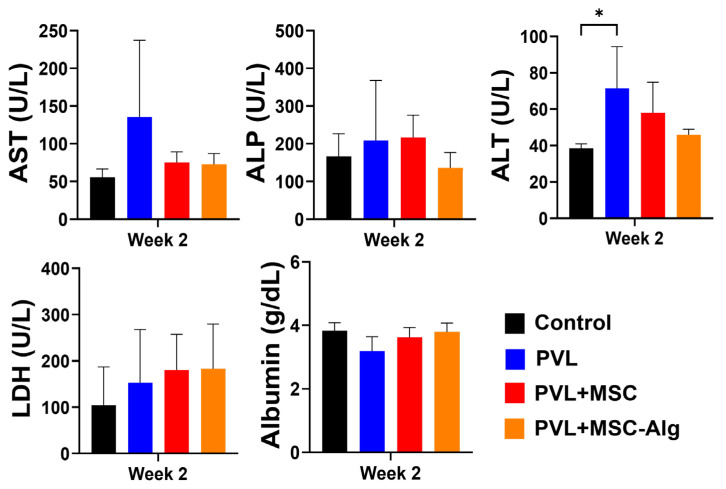
Serum chemistry levels of liver function markers among different treatment groups. The levels of AST, ALP, and ALT that are indicative of liver injury were reduced. Meanwhile, LDH and albumin levels were comparable among the different treatment groups. Abbreviations: PVL, portal vein ligation; AST, aspartate aminotransferase; ALP, alkaline phosphatase; ALT, alanine aminotransferase; LDH, lactate dehydrogenase; * *p* < 0.05.

Liver function tests were performed to correlate with the observed gross anatomical changes in the liver. Intraportal administration of MSCs loaded in alginate hydrogels resulted in improvements of AST, alkaline phosphatase (ALP), and ALT levels in the liver. The analysis revealed a notable decreasing trend in the levels of AST (*p* = 0.177), ALP (*p* = 0.587), and ALT (*p* = 0.013) in all treatment groups, although this was not statistically significant (Figure 4). The group treated with MSC-loaded hydrogels demonstrated the most pronounced improvement in serum chemistry levels (AST: 72.75 ± 14.17 U/L, ALP: 135.67 ± 41.20 U/L, ALT: 46.00 ± 2.94 U/L). In contrast, the levels of lactate dehydrogenase (LDH; control: 104.50 ± 71.37 U/L, PVL: 152.50 ± 99.78 U/L, PVL+MSC: 180.33 ± 70.18 U/L, PVL+MSC-Alg: 182.67 ± 79.20 U/L) and albumin (control: 3.83 ± 0.22 g/dL, PVL: 3.19 ± 0.39 g/dL, PVL+MSC: 3.62 ± 0.29 g/dL, PVL+MSC-Alg: 3.79 ± 0.24 g/dL) in the treatment groups were comparable to the control group and not statistically significant (LDH: *p* = 0.596, Alb: *p* = 0.061). Liška et al. reported that the administration of MSCs led to maximal growth and improvement in liver biochemical parameters [50]. Another study reported recovery of liver enzyme levels after transplantation of MSCs [51]. Furthermore, one study reported that the MSC treatment can aid in the restoration of normal liver parameters from a fibrotic, diseased state [52]. In this regard, our study displayed similar improvements, especially in the groups receiving MSCs with the alginate hydrogel.

### 2.3. Histopathological Analysis of Liver Samples After Treatment

**Figure 5:**
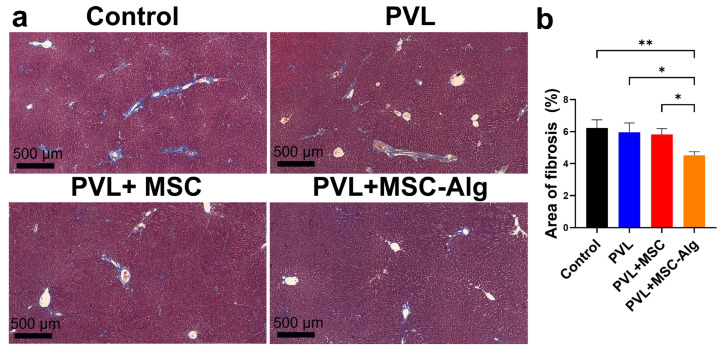
The effect of MSC-loaded hydrogels on collagen deposition in the liver. (**a**) Masson’s trichrome staining of the livers among the treatment groups showed reduction in the degree of fibrosis. (**b**) Quantification of the fibrotic areas through image analysis software revealed significant reduction in areas of fibrosis after MSC treatment. Abbreviations: Alg, alginate; MSC, mesenchymal stem cell; PVL, portal vein ligation; * *p* < 0.05; ** *p* < 0.01.

To evaluate the histopathological changes in the liver microarchitecture, trichrome staining was performed (Figure 5a). Collagen staining in the control group was greater than the treatment groups. Furthermore, periportal fibrosis with septal formation was more obvious in the control and PVL groups compared to PVL+MSC and PVL+MSC-Alg. Among the treatment groups, the PVL+MSC-Alg (4.52 ± 0.22%) group exhibited the greatest decrease in fibrotic areas compared to the control (6.23 ± 0.51%), PVL (5.96 ± 0.59%), and PVL+MSC (5.82 ± 0.38%) groups (Figure 5b). The areas of septal formation in the same group were also visually decreased, suggesting a possible decrease in the histological grade of fibrosis. Meier and colleague transplanted MSCs using alginate-based microspheres and found a similar reduction in liver fibrosis [31]. Another study [53] also showed the anti-fibrotic effect of MSC-loaded hydrogels after intraperitoneal administration in cirrhotic rats. The decrease in fibrotic markers may suggest disease resolution and tissue remodeling. Several mechanisms reported in the literature include immunomodulation by transforming growth factor-beta (TGF-β) suppression, ECM remodeling, and pro-regenerative signaling [54,55,56].

**Figure 6:**
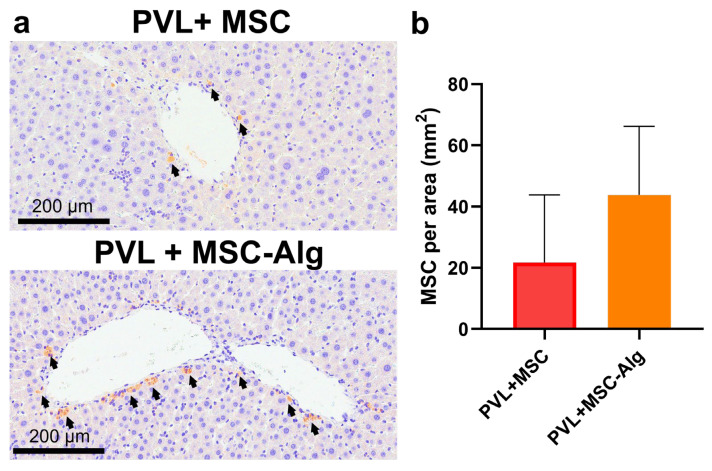
Immunohistochemistry staining of red fluorescent protein (RFP)-positive cells in the liver. (**a**) Tissue sections of rat livers treated with MSCs only (top) and MSC-loaded hydrogels (bottom) showed the presence of RFP+ cells in the periportal areas (black arrows). (**b**) Quantification of RFP+ cells demonstrated an increase in the number of RFP+ cells in groups treated with PVL+MSC-Alg (*p* = 0.0658). Abbreviations: Alg, alginate; MSC, mesenchymal stem cell; PVL, portal vein ligation; RFP, red fluorescent protein.

To confirm the retention of MSCs in the liver, immunohistochemistry (IHC) was performed to detect red fluorescent protein-MSCs (RFP-MSCs). The stained MSCs, shown as a brown stain with black arrows, reside in the periportal areas (Figure 6). Compared to the PVL+MSC group, the PVL+MSC-Alg group showed increased RFP+ staining (PVL+MSC: 21.7 ± 22.1 MSC/mm^2^ vs. PVL+MSC-Alg: 37.3 ± 16.1 MSC/mm^2^), although it was not statistically significant (*p* = 0.0658). This suggests that the hydrogel matrix enhanced cell retention, which prevented the rapid washout of cells. The hydrogel may have acted as a mechanical barrier to hold the MSCs in the liver and provide a site for cell attachment. Then, MSCs can exert more of their therapeutic effect, optimally resulting in higher liver mass regeneration and improvements in liver function markers [57,58]. Additionally, the hydrogels may have allowed the MSCs to interact and integrate more into the liver microenvironment. MSCs can transdifferentiate to hepatocyte-like cells that aid in repopulating the liver [59]. The biological cues conferred by hydrogel materials to MSCs should be investigated in future research. Understanding this will be important in devising hydrogel biomaterials with ideal physicochemical properties for supporting MSC therapy.

This study provides insights into the role of hydrogels to enhance MSC functionality in liver growth and fibrosis attenuation, though it has several limitations. While hydrogel biocompatibility was confirmed, the underlying cell–hydrogel interactions were not yet fully elucidated. The hydrogels used in the study served as a good delivery system, but alternative cell-loading strategies such as microencapsulation [60], spray drying [61,62], and 3D bioprinting [63] may offer more precise and customizable constructs. Also, investigating MSC retention and differentiation within the liver microenvironment is essential, with techniques such as genetic lineage tracing and single-cell RNA sequencing providing insights into cell fate and behavior. In vitro co-culture systems offer a platform to study MSC–hepatocyte interactions, revealing their functional relationship within the liver context. Moreover, the animal models developed F2–F3 fibrosis, which may not entirely replicate the spectrum of liver fibrosis in humans, where variability in severity is common. Therefore, future preclinical studies in large animal models and clinical trials are warranted. Nonetheless, the findings on the therapeutic benefits (i.e., enhanced liver growth, reduced fibrosis, and improved MSC retention) of MSC-loaded hydrogels are anticipated to be translatable to human applications. Further, optimization of the hydrogel, including standardization of material purification and MSC-loading protocols in accordance with clinical good manufacturing practice (GMP) standards, should be performed. Additionally, comprehensive safety and tolerability assessments must be established prior to clinical translation. All in all, our study serves as a proof of concept, illustrating that the hydrogel matrix significantly prolonged MSC retention and enhanced their effect on liver growth and fibrosis reduction.

The findings of this study will allow future researchers to explore the use of hydrogel-based cell delivery systems in augmentative cell therapy for liver regeneration and fibrosis. Our results imply that the use of cell delivery systems increases the therapeutic effect of MSCs. The MSC-loaded alginate hydrogels can also be expanded to other fibrosis-related diseases to explore their role in advancing current treatment strategies. The development of hydrogels may potentially be expanded to gene therapy [64] and antimicrobial [65] research. Identifying the exact mechanisms of biomaterial–cell interactions and the therapeutic molecules secreted by MSCs will also shed light on the future development of specific and acellular therapeutic hydrogel delivery systems. Studying optimal cell delivery strategies to maintain and promote cell function, growth, and delivery will allow the maximum potential of cell therapy to be reached in liver regeneration and other diseases.

## 3. Conclusions

**Figure 7:**
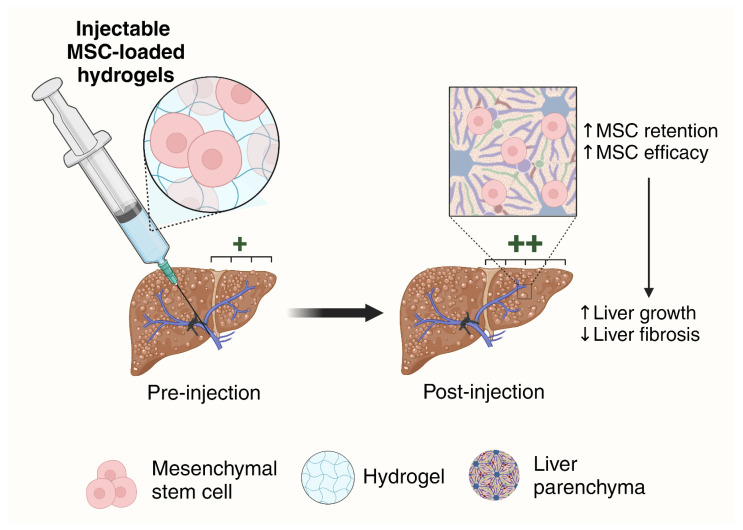
Summary of the study. The therapeutic potential of mesenchymal stem cells (MSCs) is hindered by rapid washout of cells in the liver. In this study, MSCs were loaded in injectable hydrogels and delivered in vivo. Upon injection of hydrogels, MSCs had increased retention and efficacy, which led to enhanced liver growth and attenuation of fibrosis (created with Biorender.com). Abbreviations: MSC, mesenchymal stem cell.

The impact of MSC therapy in liver diseases is important for patients who typically have severe diseases and require sufficient FLR growth. Achieving the maximum therapeutic potential of MSCs depends on their delivery and retention in the liver. In this study, we developed injectable MSC-loaded alginate hydrogels that improved liver growth and attenuated fibrosis (Figure 7). Alginate hydrogels supported MSC viability and allowed sustained cell release over 14 days. Moreover, alginate hydrogels effectively enhanced the retention and efficacy of MSCs in improving liver volume, weight, function, and fibrotic markers of CCl_4_-treated rats. This approach holds significant potential for improving outcomes in patients who are unable to undergo surgery due to insufficient liver remnant growth, offering a promising therapeutic strategy for liver regeneration and the treatment of liver diseases.

## 4. Materials and Methods

### 4.1. Materials

Sodium alginate (CAS-No. 9005-38-3), CaCl_2_ (>97%), PBS at pH 7.4, sodium citrate (ACS reagent, ≥99.0%), CCl_4_ (anhydrous, ≥99.5%), olive oil (highly refined, low acidity), trypan blue solution (0.4%), Dulbecco’s modified eagle medium-high glucose (DMEM), fetal bovine serum (FBS, Gibco, Waltham, MA, USA), penicillin-streptomycin (10,000 U/mL), and neutral buffered formalin (10%) were obtained from Sigma-Aldrich (St. Louis, MO, USA). Iohexol (Omnipaque, 350 mgI/mL, GE Healthcare, Chicago, IL, USA) was the contrast agent for imaging studies.

### 4.2. Cell Culture and Animals

Bone marrow-derived Sprague–Dawley RFP-MSCs were acquired from Creative Bioarray (Shirley, NY, USA). A homogenous population of RFP-MSCs (passage 2) was cultured in the complete medium of DMEM, 10% (*v*/*v*) FBS, and 1% (*v*/*v*) penicillin-streptomycin. Cells were incubated at 37 °C in a humidified incubator atmosphere with 5% CO_2_. The media was changed every 2–3 days. All experiments used cells from passage 3–5.

All animal experiments were carried out under the approval of The University of Texas MD Anderson Cancer Center Institutional Animal Care and Use Committee (Protocol No: 2301-RN01). Female Sprague–Dawley (SD) rats (400 SAS SD, Charles Rivers Laboratories, Wilmington, MA, USA) at age 6–8 weeks with an average weight of 150–200 g were used for the experiments. The animals were placed in a sterile enclosure at a temperature of 22 ± 4 °C and relative humidity of up to 60%. Daytime and night were provided in a 12 h light/dark cycle with free access to food and water ad libitum. The total number of animals in the study was 24, which were divided into four treatment groups.

### 4.3. Preparation of MSC-Loaded Hydrogels

The MSC-loaded hydrogels were prepared by combining a suspension of MSCs in alginate solution. Sodium alginate (4–12 cP, viscosity) was dissolved in sterile ultrapure water to prepare 1.5% (*w*/*v*) alginate aqueous solution under continuous stirring for 30 min at room temperature. Then, the alginate solution was sterile-filtered using a 0.22 µm filter. The RFP-MSCs were loaded onto the alginate solution by mixing the cell suspension with the hydrogel solution to achieve a final cell density of 5 × 10^6^ cells/mL. The resulting MSC-loaded alginate was mixed with 50 mM CaCl_2_ in deionized water at a ratio of 1:1 to initiate cross-linking between alginate and the calcium ions. To ensure stable hydrogel formation, the hydrogels were kept in CaCl_2_ for ten minutes (at room temperature) before removing the excess CaCl_2_. The solidified MSC-loaded hydrogels were immediately placed in media at 37 °C and cultivated. The MSC-loaded alginate hydrogels were then immediately used for subsequent in vitro studies.

The swelling capacity of the hydrogels was assessed using the standard gravimetric method. Hydrogel samples measuring 1 × 0.5 cm were initially weighed and then incubated at 37 °C in distilled water (50 mL). After 24 h of incubation, the excess liquid was removed, and the samples were re-weighed. The degree of swelling was calculated as follows:

% swelling = ((Weight_24h_ − Weight_dry_))/Weight_dry_) × 100% (1)

### 4.4. In Vitro MSC Survival and Release in Hydrogels

To determine the cell viability after loading in alginate, the MSC-loaded hydrogels were placed into 12-well plates and monitored for two weeks. At the designated time points, the MSC-loaded hydrogels were incubated in 3.2% sodium citrate for 5 min at 37 °C. A volume of the released cells was collected, and an equal volume of trypan blue dye was added. The suspension was then loaded into a Cellometer K2 Image Cytometer Automated Cell Counter (Nexcelom Bioscience, Lawrence, MA, USA) for percent (%) cell viability measurement.

The hydrogels were incubated in media with PBS matched with physiologic phosphate levels to monitor the release of cells from the hydrogels for two weeks. Briefly, MSC-loaded hydrogels (500 µL) were placed on the upper chamber of a 50 µm cell strainer and positioned in a 12-well plate. Then, 3 mL of media with PBS was added to the well, ensuring that the hydrogels were completely submerged. The released cells at the bottom of the well were collected and counted. Meanwhile, the unreleased cells were transferred to a different plate and dissolved with 3.2% sodium citrate. After gel dissolution, the remaining cells were counted. The percent (%) cell release was calculated as follows:

%Cell release = (released cells)/(released + unreleased cells) × 100%(2)

### 4.5. Cirrhosis Induction and Portal Vein Ligation in Rats

To investigate the effect of direct portal vein injection of MSC-loaded hydrogels in vivo, liver cirrhosis was induced in SD rats. CCl_4_ in olive oil (1:1) was injected intraperitoneally at dose of 1 mL/kg twice weekly for 8 weeks. The rats were weighed before injection for dose adjustment. The rats (*N* = 24) were randomly assigned and evenly distributed among four experimental groups: control (negative), PVL, PVL+MSC, and PVL+MSC-Alg.

All surgical procedures were performed under anesthesia with isoflurane (1.5%, Covetrus, Portland, ME, USA) and oxygen (flow rate, 0.5 L/min). In the PVL groups, selective ligation of the left portal vein was performed after a mini-laparotomy. After careful dissection, the left portal vein was ligated with a 4-0 non-absorbable vicryl suture (Ethicon, Raritan, NJ, USA), while the right portal vein, hepatic arterial circulation, and biliary duct branches were preserved. The control rats had no treatment. For the MSC group, 1 × 10^6^ cells were administered at 200 µL. For the MSC-Alg group, the MSC-loaded hydrogels with 1 × 10^6^ cells, at the same volume as the MSC group, were utilized. The rat liver volume, weight, and function were then evaluated for 2 weeks. At the end of the experiments, the rats were anesthetized with isoflurane and euthanized. Serum samples were collected for every rat. Liver tissues were also harvested and fixed in 10% neutral buffered formalin.

### 4.6. Monitoring of Liver Volume, Weight, and Function

After surgery, the liver volumes of all animals were monitored weekly for 2 weeks. The liver volumes were imaged using a Siemens SOMATOM Definition Edge scanner (Siemens Healthineers, Erlangen, Germany). A bolus injection of iohexol at a dose of 170 mg/kg was used to enhance contrast in the liver. To estimate liver volumes, Medical Imaging Modeling (MIM) software (MIM Software Inc., Cleveland, OH, USA) was used. MIM software provided tools for liver lobe segmentation and quantification of the liver volumes. The growth by volume of the non-ligated liver lobes relative to the total liver was calculated using the following formula:

FLR ratio growth (volume) = (right liver volume)/(total liver volume)(3)

Serum chemistry and necropsy were also performed to support the imaging findings. Prior to sacrifice, rat sera were collected, and levels of AST, ALP, ALT, LDH, and albumin were quantified using an automatic biochemical analyzer. Upon necropsy, the liver samples were inspected for abnormalities and divided into left (left and middle) and right (right and caudate). The weights of each liver section were measured. The growth by weight of the non-ligated liver lobes relative to the total liver was calculated using the following formula:

FLR ratio growth (weight) = (right liver weight)/(total liver weight)(4)

### 4.7. Histopathological Examination

The fixed liver samples were processed and embedded in paraffin in preparation for histopathologic examination. Then, the processed samples were sectioned at a thickness of 5 µm. To evaluate the extent of hepatic fibrosis, collagen deposition in the liver tissue was evaluated by Masson’s trichrome staining. Furthermore, IHC staining for the anti-RFP was performed to detect and quantify the presence of the injected RFP-MSCs. An Aperio LV1 real-time digital pathology system (Leica Biosystems, Buffalo Grove, IL, USA) was used to scan and capture the images from the stained slides. For quantification of the percent (%) fibrotic areas and RFP+ cells, the HALO image analysis platform (Indica Labs, Albuquerque, NM, USA) was utilized.

### 4.8. Statistical Analysis

GraphPad Prism software, version 10.0.3 (GraphPad, San Diego, CA, USA) was used for statistical analyses. The experimental data were expressed as means ± standard deviations. The studied responses were evaluated using a two-tailed *t*-test or analysis of variance (ANOVA) where appropriate. The threshold for statistical significance was defined as *p* < 0.05.

## Data Availability

The original contributions presented in this study are included in the article/Supplementary Materials. Further inquiries can be directed to the corresponding author.

## Supplementary Materials

The following supporting information can be downloaded at https://www.mdpi.com/article/10.3390/gels11040250/s1. Figure S1: Swelling study of the alginate hydrogels with and without MSCs in sterile water for 24 h.
