# Supplementary material for: Mesenchymal Stem Cells Loaded in Injectable Alginate Hydrogels Promote Liver Growth and Attenuate Liver Fibrosis in Cirrhotic Rats
Source: Gels. 2025 Mar 27;11(4):250. doi: 10.3390/gels11040250 (PMC12027234; doi:10.3390/gels11040250)
Supplement: Supplementary file 1 [file gels-11-00250-s001.zip › gels-3532172-supplementary.pdf]

SUPPLEMENTARY FIGURE

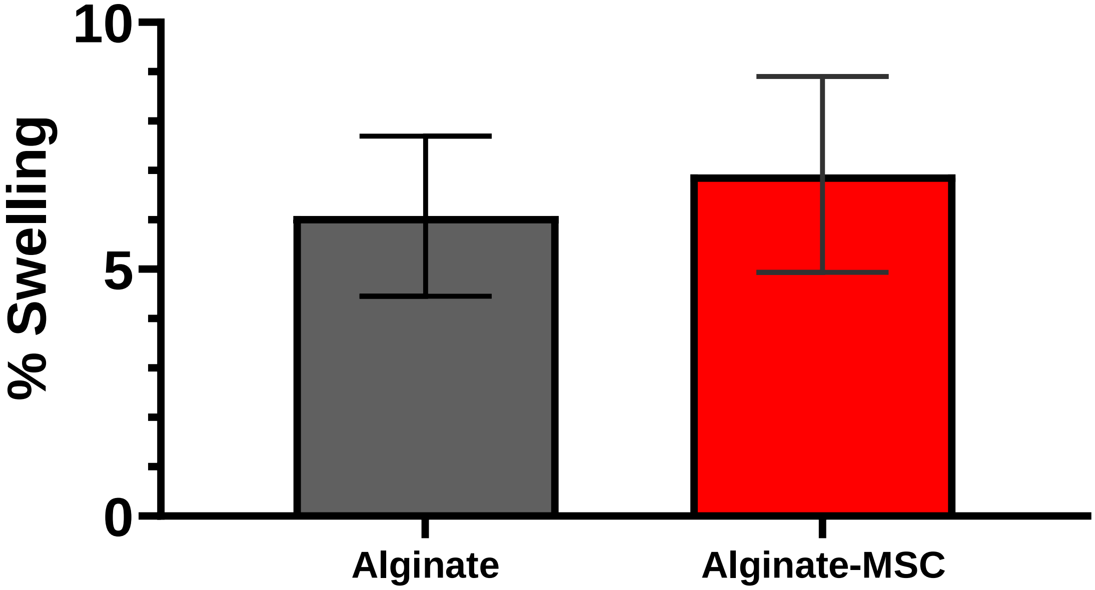

Supplementary Figures S1. Swelling study of the alginate hydrogels with and without MSCs in sterile water for 24 hours. The swelling behavior of alginate hydrogels, both with and without mesenchymal stem cells (MSCs), was evaluated in sterile water over a 24-hour period. A total volume of 1 mL of formed alginate hydrogels was immersed in sterile water, and their dry weights were recorded prior to and following the 24-hour soaking period. The hydrogels without MSCs exhibited a swelling ratio of 6.08 ± 1.62%, whereas those containing MSCs demonstrated a swelling ratio of 6.92 ± 1.98% ($p=0.802$).
